# “Fontan Conduit Stent-Angioplasty and Progression of Fontan-Associated Liver Disease”

**DOI:** 10.1007/s00246-024-03426-8

**Published:** 2024-03-01

**Authors:** Umakanthan Kavin, Aniqa Shahrier, Varsha M. Bandisode, Shahryar M. Chowdhury, John F. Rhodes, Stephanie S. Gaydos

**Affiliations:** 1 Division of Pediatric Cardiology, Department of Pediatrics, Children’s Hospital Colorado, Aurora, CO 80045 USA; 2 Division of Pediatric Cardiology, Department of Pediatrics, Pediatrix Cardiology Associates and Tampa Bay Adult Congenital Heart Center, Tampa, FL 33607 USA; 3 Division of Pediatric Cardiology, Department of Pediatrics, Medical University of South Carolina, Charleston, SC 29425 USA

**Keywords:** Fontan, Stent angioplasty, Liver disease

## Abstract

Patients with Fontan circulation insidiously develop congestive hepatopathy related to chronically reduced cardiac output and central venous hypertension, also known as Fontan-associated liver disease (FALD). Fontan pathway obstruction is increasingly detected and may accelerate FALD. The impact of conduit stent angioplasty on FALD is unknown. Retrospective, single-center review of patients with Fontan circulation who underwent conduit stent angioplasty at cardiac catheterization over 5-year period. Demographics and cardiac histories were reviewed. Labs, liver ultrasound elastography, echocardiogram, hemodynamic and angiographic data at catheterization were recorded pre- and post-stent angioplasty. Primary outcome was change in hepatic function via MELD-XI scores and liver stiffness (kPa), with secondary outcomes of ventricular function, BNP, and repeat catheterization hemodynamics. 33 patients underwent Fontan conduit stent angioplasty, 19.3 ± 7.0 years from Fontan operation. Original conduit diameter was 19.1 ± 1.9 mm. Prior to angioplasty, conduit size was reduced to a cross-sectional area 132 (91, 173) mm^2^ and increased to 314 (255, 363) mm^2^ post-stent. Subjects’ baseline median MELD-XI of 11 (9, 12) increased to 12 (9, 13) at 19 ± 15.5 months post-angioplasty (*n* = 22, *p* = 0.053). There was no significant change in liver stiffness at 12.1 ± 8.9 months post-angioplasty (*n* = 15, *p* = 0.13). Median total bilirubin significantly increased (1.4 [0.9, 1.8]), from baseline 1.1 [0.7, 1.5], *p* = 0.04), as did median BNP (41 [0, 148] from baseline 34 [15, 79]; *p* = 0.02). There were no significant changes in ventricular function or repeat invasive hemodynamics (*n* = 8 subjects). Mid-term follow-up of Fontan subjects post-conduit stent angioplasty did not show improvements in non-invasive markers of FALD.

**Supplementary Information:**

The online version contains supplementary material available at 10.1007/s00246-024-03426-8.

## Introduction

Since being introduced in 1971, the Fontan palliation has served as the final surgical step in the single ventricle pathway for many different congenital cardiac pathologies^1^. As the surgical techniques have continued to evolve in addition to advancements in medical management, patients with Fontan physiology have continued to live longer, most well into adulthood. There are, however, many complications associated with the Fontan physiology including arrhythmia, thromboembolism, ventricular failure, kidney disease, protein-losing enteropathy, and liver fibrosis or Fontan-associated liver disease (FALD) [1]. FALD remains clinically important especially when evaluating for transplant eligibility as it may necessitate combined heart-liver transplant versus heart transplant alone. While there is still much to explore regarding the pathophysiology, it is thought that a large contributor to FALD is persistently elevated central venous pressures, loss of venous pulsatility, and marginal cardiac output which can lead to cardiac cirrhosis [1]. The development of FALD is insidious and is difficult to diagnose as the majority of patients will be asymptomatic without significant physical or laboratory abnormalities [1, 2]. A complication that may exacerbate FALD is Fontan conduit stenosis, which may compromise the hemodynamics of the conduit and lead to more severe central venous congestion, particularly proximal to the obstruction. Fontan conduit stenosis occurs at a considerable rate with a mean decrease of cross-sectional area (CSA) of 14% at 3 years for extracardiac conduits [3]. Significant stenosis can be defined as a reduction of CSA of 25% or a measurable gradient across the conduit, which can occur in approximately 22% of patients [4]. This may even be an underestimate at present, as patients followed longitudinally in the current era have more standardized imaging assessments of the Fontan conduit compared to in the past. Fontan stent angioplasty to relieve conduit stenosis has already been shown to improve various comorbidities of Fontan circulation, including NYHA class, exercise capacity, severity of protein-losing enteropathy (PLE), severity of ascites, and pulmonary vascular resistance (PVR) [4–9]. Several studies have also demonstrated that higher Fontan and central venous pressures are often associated with more advanced histologic liver fibrosis and liver stiffness [10–12]. The aim of this study was to evaluate whether non-invasive markers of FALD change following treatment of Fontan conduit stenosis via stent angioplasty. We hypothesized that stent angioplasty would reduce hepatic congestion, and thus hinder or even improve the progression of FALD.

## Methods

This retrospective, single-center study included all patients with Fontan physiology who had undergone Fontan conduit stent angioplasty at the Medical University of South Carolina. Our institutional catheterization record database was searched to identify the subject group that underwent this intervention between January 2017 and August 2022. Subjects’ medical records were reviewed for demographics, primary cardiac lesion, age at Fontan operation, type of Fontan repair, and extracardiac conduit (ECC) size if applicable. Chart review was performed to obtain the most proximal pre-intervention FALD markers, defined as serum hepatic biomarkers, Model for End-stage Liver Disease excluding INR (MELD-XI) scores, liver elastography stiffness (kPa), and liver biopsies if available. Pre-intervention echocardiograms were reviewed for assessments of ventricular function and atrioventricular valve regurgitation (AVVR). The cardiac catheterization record was reviewed to obtain baseline hemodynamics, cross-sectional area (CSA) of the Fontan conduit by angiography before and after stent deployment, type of stent utilized, and post-stent deployment hemodynamics. At a minimum of three months post-procedure, the same outcome markers were recorded at most proximal point to time of chart review (outside of hospitalization for decompensated heart failure and/or immediately prior to orthotopic heart transplant). Any available repeat cardiac catheterization hemodynamics and stented-conduit CSA measured by catheterization angiography or computerized tomography (CT) scan were also reviewed. The project was approved by the MUSC Institutional Review Board.

## Statistics

The distribution of data was tested using the Shapiro–Wilk test. Data are reported as mean ± standard deviation for normally distributed data or median (interquartile range) for non-normally distributed data. Differences between pre- and post-stent variables were tested using Wilcoxon Signed-Rank test or the McNemar test as appropriate. A *p* value less than 0.05 was considered statistically significant. All statistics were performed using IBM SPSS Statistics software v. 27 (manufactured in Armonk, NY).

## Results

A total of 33 Fontan patients underwent conduit stent angioplasty (52% males) within the study period, with demographic data summarized in Table 1. Indications for cardiac catheterization were surveillance hemodynamic assessment for the majority of subjects, and several others referred due to a clinical change. Patients were a median age of 3.7 years (IQR 3.0, 5.1) at the time of their Fontan operation. The majority of the cohort had an extracardiac conduit (ECC) (*n* = 23, 70%) with the remaining patients having a lateral tunnel. Median ECC size at surgery was 18 mm (IQR 18, 20). Patients’ pre-stent angioplasty median MELD-XI score was 11.5 (IQR 9.0, 12.0) (*n* = 32). Baseline liver elastography data were available for 23 of 33 patients prior to undergoing stent angioplasty, with median liver stiffness velocity of 2.0 m/s (IQR 1.7, 2.3) correlating to a liver stiffness of 12.0 kPa (IQR 9.0, 15.2). Three subjects had prior pre-stent liver biopsies ranging from grade 1 to 4 fibrosis on pathology. Most proximal pre-stent angioplasty echocardiogram showed the majority of patients had normal to mildly depressed ventricular function (*n* = 25, 76%) and normal or mild AVVR (n = 26, 79%).

**Table 1:** Demographic Patient Information (*n* = 33)

Male sex, *n* (%)	17 (52%)
Age at Fontan (years)	3.7 (3.0, 5.1)
Ventricular dominance, *n* (%)	
Right	14 (42%)
Left	15 (46%)
Indeterminate	4 (12%)
Fontan type, *n* (%)	
Lateral tunnel	10 (30%)
Extracardiac conduit	23 (70%)
ECC conduit size, *n* (%)	
16 mm	1 (4%)
18 mm	10 (44%)
20 mm	7 (30%)
22 or 23 mm	3 (13%)

Demographics and cardiac history of the subject sample who underwent Fontan conduit stent angioplasty. Data reported as median (interquartile range) or count (percentage). *ECC* extracardiac conduit

### Cardiac Catheterization Data During Stent Angioplasty

**Fig. 1:**
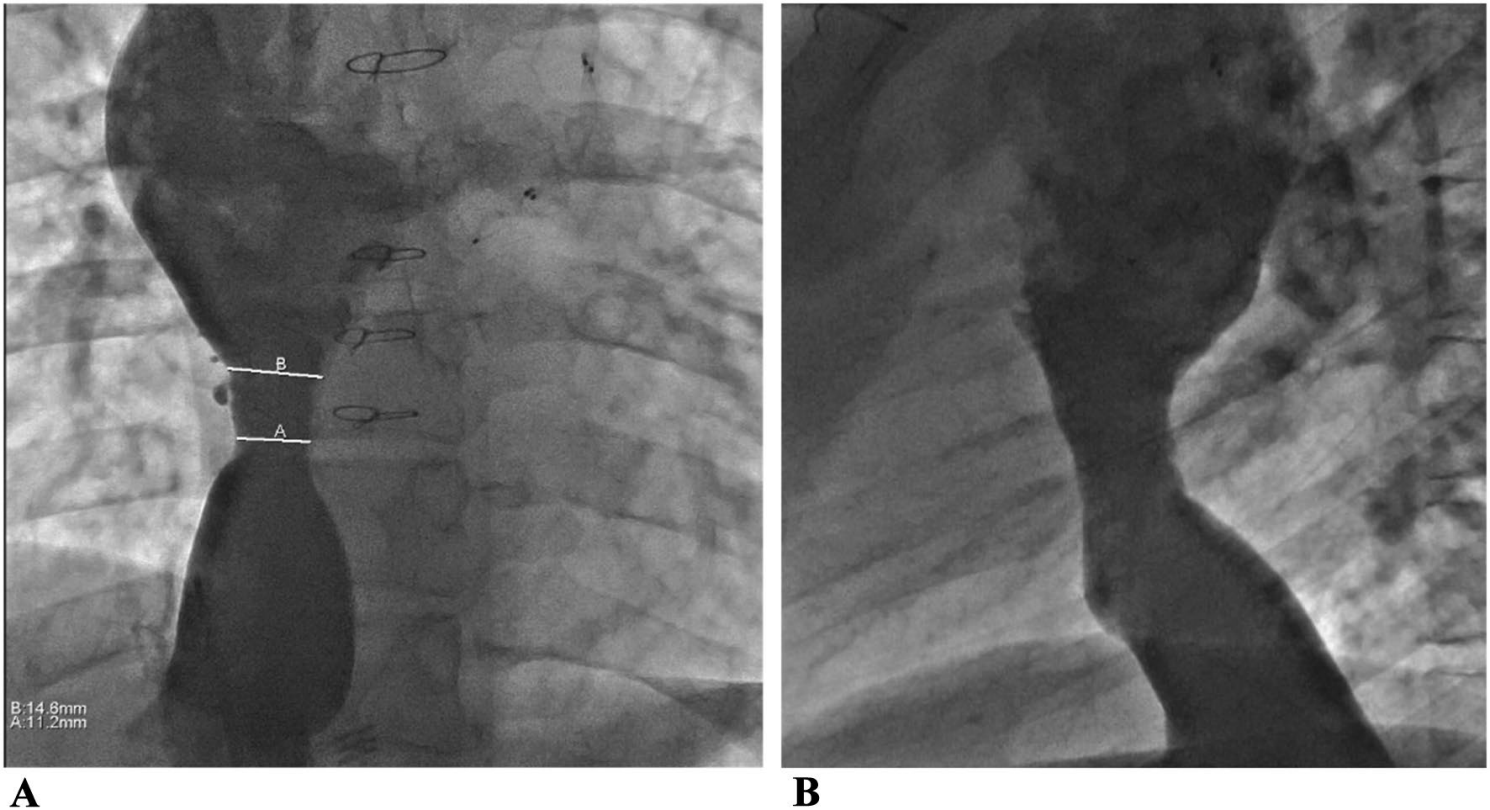
Angiographic views of a patient with double-inlet left ventricle and l-transposition of the great arteries who underwent an intra-atrial lateral tunnel Fontan procedure now with focal Fontan stenosis and aneurysmal dilation of the vena cava superiorly and inferiorly, prior to stent-angioplasty. **A** anteroposterior view, **B** lateral view

The average age at conduit stent angioplasty was 23.8 ± 8.0 years, at an average of 19.3 ± 7 years from Fontan operation. Measurements of the inferior vena cava (IVC), Fontan conduit, and superior vena cava (SVC) at cardiac catheterization were performed in both posteroanterior (PA) and lateral projections on images with contrast (Fig. 1). After stenting, the same diameters were measured again with calculations of pre- and post- angioplasty conduit CSA. Pre-stent angiography showed a median minimal Fontan conduit posteroanterior diameter of 12.6 mm (IQR 9.9, 14.0), minimal lateral diameter of 14.0 mm (IQR 10.5, 16.2), and the minimal Fontan CSA of 132 mm^2^ (IQR 91, 173). The median SVC, Fontan, IVC, and mean pulmonary artery pressures were 15 mmHg (IQR 12, 18) and pulmonary capillary wedge pressures 10 mmHg (IQR 8, 11); median transpulmonary gradient was 5.0 mmHg (3.0, 6.5). The median cardiac index was 2.54 L/min/m^2^ (IQR 2.31, 3.25), and the mean pulmonary vascular resistance index was 2.06 WUm^2^ (IQR 1.61, 3.40). Subject group hemodynamic data and procedural measurements are summarized in Table 2**.**

**Table 2:** Hemodynamic and Angiographic Characteristics at Time of Stent Angioplasty (*n* = 33)

Age at stent angioplasty (years)	23.8 ± 8.0
Duration from Fontan (years)	19.3 ± 7.0
Pre-intervention measurements	
Posteroanterior Fontan minimal diameter (mm)	12.0 (9.9, 14.0)
Lateral Fontan minimal diameter (mm)	14.0 (10.5, 16.2)
Minimal Fontan CSA (mm^2^)	131.9 (91.2, 173.2)
Largest balloon used for dilation (mm)	20.0 (20.0, 23.0)
SVC pressure (mmHg)	15.0 (12.0, 17.5)
Fontan pressure (mmHg)	15.0 (13.0, 17.5)
IVC pressure (mmHg)	15.0 (13.0, 18.0)
Mean pulmonary artery pressure (mmHg)	15.0 (12.0, 17.0)
Mean pulmonary capillary wedge pressure (mmHg)	10.0 (8.0, 11.0)
Transpulmonary gradient pressure (mmHg)	5.0 (3.0, 6.5)
Cardiac index (L/min/m^2^)	2.54 (2.31, 3.25)
Pulmonary vascular resistance index (WUm^2^)	2.06 (1.61, 3.40)
Post-Intervention measurements	
Final PA diameter (mm)	20.0 (18.0, 21.0)
Final lateral diameter (mm)	20.0 (18.0, 22.0)
Final CSA (mm^2^)	314 (255, 363)
SVC pressure (mmHg)	16.0 (12.8, 20.0)
Fontan pressure (mmHg)	15.0 (12.5, 18.0)
IVC pressure (mmHg)	16 (13.0, 20.0)

Interventional data of subjects who underwent Fontan conduit stent angioplasty. Data are reported as means, ± standard deviation, or medians (interquartile ranges)

*CSA* cross-sectional area, *SVC* superior vena cava, *IVC* inferior vena cava, *CVP* central venous pressure, *PCW* capillary wedge pressure

A variety of stent sizes and types were utilized for conduit stent angioplasty with a range of maximum balloon expansion size from 10 to 28 mm (Supplemental Appendix 1*).* Conduit stenting was successful in all subjects without complications. Following stent angioplasty, the dimensions of the Fontan conduit increased with a median PA diameter of 20.0 mm (18.0, 21.0), lateral diameter of 20.0 mm (18.0, 22.0), and a CSA of 314 mm^2^ (255, 363). The immediate post-stent hemodynamics were minimally changed, with a median SVC pressure of 16.0 mmHg (12.8, 20.0), Fontan pressure of 15 mmHg (12.5, 18.0), and IVC pressure of 16 mmHg (13, 20). Post-stent cardiac output indices were not uniformly re-calculated. Of note, 13 subjects underwent concurrent interventions for other abnormal findings noted at time of catheterization for Fontan conduit stent angioplasty: 8 collateral closure, 3 other stent angioplasty (coarctation, left pulmonary artery, and iliac vein), and 2 fenestration interventions (closure for 1 and stent balloon angioplasty for 1).

### Mid-Term Outcomes post-Stent Angioplasty

**Fig. 2:**
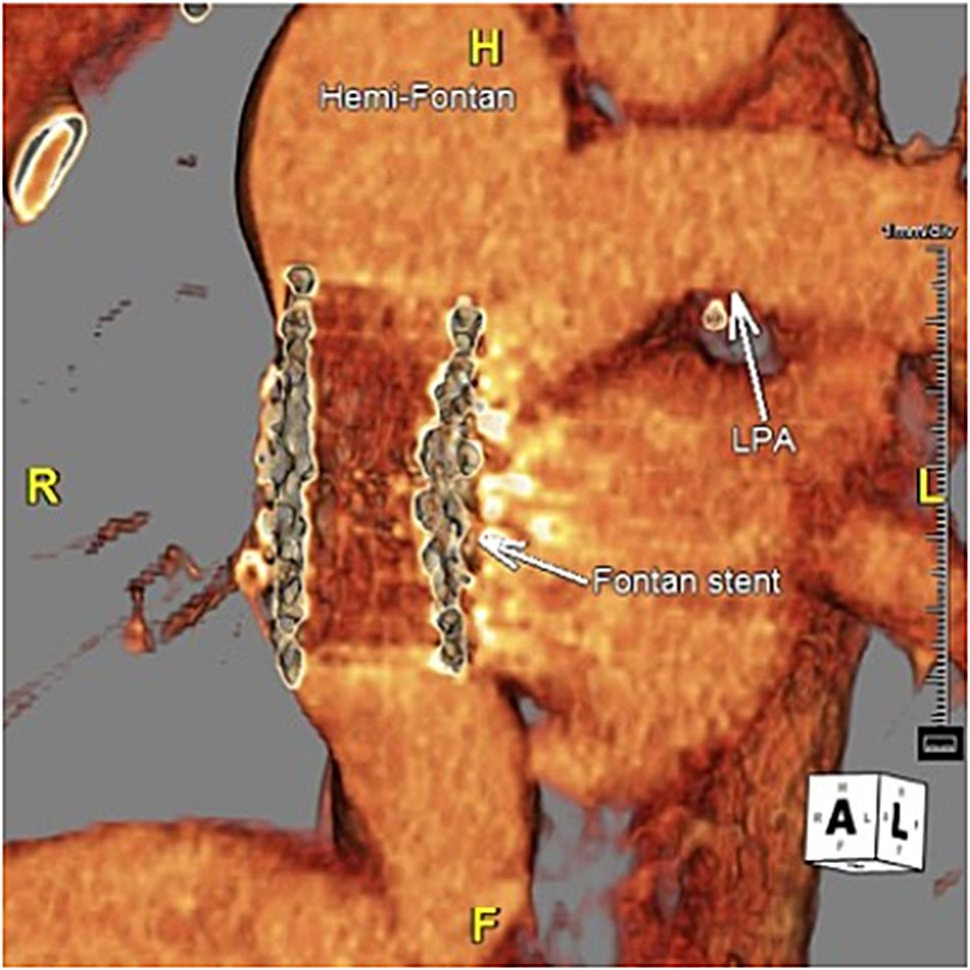
CTA of a patient one year following lateral tunnel Fontan conduit stent angioplasty, showing patent stent and unobstructed Fontan pathway

Twenty-two subjects (67% of cohort) had paired pre- and post-angioplasty labs to compare median MELD-XI scores, with average reassessment at 19 ± 15.5 months post-angioplasty. Subjects’ baseline MELD-XI score was 10.5 (9.0, 12.0) and slightly increased on reassessment to 11.5 (9.0, 13.0) (*p* = 0.053). The median post-angioplasty total bilirubin significantly increased from baseline 1.1 (0.7, 1.5) to 1.4 (0.9, 1.8) (*p* = 0.04), though there was no significant change in pre- and post- serum creatinine, platelet count, INR, serum sodium, total protein, albumin, or transaminases (Table 3). Fifteen subjects (45% of cohort) had paired pre- and post-angioplasty ultrasound elastography to reassess median liver stiffness at an average of 12.1 ± 8.9 months post-angioplasty. Baseline liver stiffness of 12.0 (9.4, 15.2) kPa showed a statistically insignificant downtrend to 10.8 kPa (9.0, 12.8) post-stent (*p* = 0.13). Only eight subjects had spleen size recorded on paired pre- and post- ultrasound reports without a significant change (*p* = 0.40). No post-angioplasty liver biopsies available. There were no hospitalizations post-angioplasty for acute liver injury or decompensated liver failure in chart review. Among 16 subjects with matching pre- and post-angioplasty BNP’s, the median baseline value 33.5 (15.2, 78.6) significantly increased to 41.0 (0, 147.8) (*p* = 0.02). There were no significant changes in echocardiographic ventricular function (*p* = 1.00, *n* = 21 subjects) or hemodynamics via repeat catheterization (*n* = 9 subjects, Wilcoxon signed rank test comparisons of median pre- and post-angioplasty pressures all > 0.05) (Table 4). Seven subjects underwent repeat CTA heart on average 1.6 years post-intervention to reassess cardiovascular anatomy and stent patency without any complications identified with the stent **(**Fig. 2**).** Within the retrospective review period, one subject died of decompensated heart failure 6.5 months post-angioplasty, and two underwent orthotopic heart transplant between 9 and 11 months post-angioplasty.

**Table 3:** Paired pre- and post- angioplasty outcomes (medians)

	Pre	Post	N	*p*-value
MELD-XI	10.5 (9, 12)	11.5 (9, 13)	22	0.053
Bilirubin	1.1 (0.7, 1.5)	1.4 (0.9, 1.7)	22	0.038
Creatinine	0.8 (0.7, 0.9)	0.8 (0.6, 1.0)	22	0.885
Liver Stiffness (kPa)	12.0 (9.4, 15.2)	10.8 (9.1, 12.8)	15	0.131
Hemoglobin	14.6 (13.7, 16.6)	15.7 (14.4, 16.6)	21	0.027
Hematocrit	43.0 (14.9, 49.0)	47.7 (44.6, 50.8)	21	0.016
Platelets	189 (120, 244)	207 (110, 232)	21	0.590
Albumin	4.1 (3.8, 4.2)	4.1 (3.9, 4.3)	22	0.183
AST	24.0 (17.8, 34.3)	25.0 (22.0, 30.0)	22	0.807
ALT	24.0 (17.0, 28.3)	23.5 (20.0, 32.0)	22	0.673
GGT	64.0 (38.5, 88.5)	58.0 (42, 108)	8	0.735
INR	1.16 (1.05, 1.29)	1.20 (1.05, 1.38)	16	0.233
BNP (ng/mL)	33.5 (15.2, 78.6)	41.0 (0, 148)	16	0.023
AFP	2.0 (2.0, 6.0)	2.3 (2.0, 4.7)	7	1.0
Spleen size	11.8 (10.8, 13.1)	12.0 (10.4, 14.3)	9	0.396
Echo: moderately or severely reduced ventricular function, n (%)	8 (38%)	9 (42%)	21	1.0
Echo: moderate or severe AVVR, n (%)	6 (29%)	6 (29%)	21	1.0

Subjects’ paired labs, liver elastography ultrasound findings, and echocardiography findings at most proximal point pre-stent angioplasty and most recent point post-stent angioplasty. Data reported as medians (interquartile range) or counts (percentage). Sample size for each paired data-point reported as (N). Statistics via Wilcoxon Signed Rank Test or McNemars test as appropriate

**Table 4:** Hemodynamics of patients with subsequent cardiac catheterization studies

No	Fontan mmHg	PCW mmHg	CI	PVRi	CVP/PCW	Years post-stent	Fontan mmHg	PCW mmHg	CI	PVRi	CVP/PCW
1	18	10	2.30	3.40	1.8	2.7	14	10	2.70	1.48	1.4
2	32	10	2.54	4.40	1.8	1.8	16	10	2.84	2.96	1.6
3	13	10	2.36	1.19	1.3	1.5	20	15	1.77	4.24	1.3
4	14	10	4.16	0.72	1.3	4.0	11	9	2.27	0.75	1.2
5	24	17	4.29	3.90	1.4	0.3	22	15	3.04	2.85	1.0
6	14	8	1.97	3.04	1.8	0.8	15	8	3.41	1.17	1.9
7	21	15	2.95	2.50	1.4	0.8	21	17	3.52	1.70	1.2
8	16	12	2.54	2.29	1.3	0.7	23	14	4.97	3.89	1.6
9	12	7	n/a	n/a	1.7	1.7	16	11	2.84	2.56	1.5

Subjects with repeat cardiac catheterizations post-stent angioplasty, comparing initial baseline hemodynamics at time of intervention and subsequent reassessment hemodynamics post-intervention. Mean post-capillary wedge pressure (PCW), cardiac index (CI) in L/min/m2, indexed pulmonary vascular resistance (PVRi) in Woods units per m2, and central venous pressure (CVP)/PCW ratio

## Discussion

Review of our single-center experience shows that Fontan stent angioplasty as treatment of conduit stenosis is safe, similar to others’ reports. Stent angioplasty did not result in improved non-invasive FALD markers at mid-term follow-up in this diverse patient group. In fact, our results suggest continued evidence of worsening liver health with a near-significant trend of increased post-angioplasty MELD-XI scores (most likely due to significantly increased total bilirubin on lab reassessments). There was not a similar trend of worsening labs thought to reflect clinically significant portal hypertension or liver dysfunction. Mean liver stiffness did not significantly change over 1-year post-angioplasty, and no patients had primary hospitalizations for decompensated liver failure or sequelae of cirrhosis. Among subjects with reassessment via cardiac catheterization, stent angioplasty did not result in consistent changes in Fontan or intra-cardiac hemodynamics.

Significant Fontan conduit pathway obstruction is an increasingly recognized late-complication in single ventricle patients, often occult in symptomatology and first identified at routine catheterization [3, 4]. This issue is intuitively problematic for success in this unique circulation, and treatment via percutaneous transcatheter stenting of the obstruction has shown promising improvements in exercise capacity and NYHA class, cyanosis, ascites, pulmonary hypertension, and protein-losing enteropathy [4–7]. These improvements presumably decrease risk of Fontan circulation-related complications, reduce or delay hospitalizations, and cost to healthcare system. Similarly intuitive is the premise that conduit obstruction may significantly contribute to FALD with increased flow resistance, compromised pathway efficiency, and greater downstream hepatic congestion. However, it has not been clearly demonstrated that treatment of pathway obstruction similarly treats FALD. This is the first study to specifically examine whether there are any changes in non-invasive FALD markers post-stent angioplasty, not identifying any clear sign of improvement.

The increased serum bilirubin noted post-angioplasty in this small sample is unclear, though may be explained by other causes of hepatic insults imposed by Fontan circulation and univentricular dysfunction. The study sample was heterogenous in baseline cardiac function, with many subjects exhibiting objective markers of Fontan and/or cardiac failure prior to stent angioplasty. Nearly a quarter of the cohort had baseline moderate or severely reduced ventricular function (*n* = 8, 24%), at least moderate AVVR (*n* = 7, 21%), and resting Fontan pressure ≥ 18 mmHg (*n* = 8, 24%). Nearly half had reduced cardiac output < 2.5 ml/kg/m2 at baseline catheterization (*n* = 15, 45%). With two subjects undergoing cardiac transplantation and one mortality within the study period, a portion of the baseline group was quite sick. Subjects with these or similar risk factors likely had closer clinical monitoring and were more represented in the sample with available post-angioplasty comparison outcomes. Relief of conduit obstruction would not be expected to completely reverse these other contributors to liver health, and hence confound these non-invasive FALD markers. A future study to specifically examine outcomes in a subject sample with similar (healthier) baseline univentricular health would be an important follow-up to this work.

Similarly, a statistically significant increase in post-angioplasty BNP was found, with reassessment available in less than half of the cohort. These subjects with available reassessments may have represented a higher-risk group with more frequent clinical monitoring due to other suboptimal circulatory factors. Additionally, the absolute median BNP values (32 pre-stent to 41 post-stent) are still quite low and unlikely reflect clinical relevance, particularly in absence of similar echocardiographic or invasive hemodynamic changes.

Overall, our results reiterate that Fontan stent angioplasty is safe and effective to treat conduit stenosis, though suggest that clinicians may not expect to see an improvement in these non-invasive FALD markers at mid-term follow-up. There is a wide spectrum of “non-invasive FALD surveillance” markers with practice divergence center-to-center. There may be optimism that our center’s sample did not demonstrate statistically significant progression in these markers of FALD over 1–2 years post-angioplasty. It remains unknown whether the intervention may temporize FALD progression compared to longstanding untreated conduit obstruction. A study utilizing matched controls diagnosed with Fontan conduit stenosis with and without stent angioplasty could best answer this question, particularly if excluding subjects with significant baseline Fontan and univentricular dysfunction to avoid confounders. However, this poses ethical challenges given other the studied benefits seen following obstruction treatment.

### Limitations

There were several limitations in this study. Study design was retrospective and observational among a single-center with a small-number cohort. Additionally, there were not uniform paired pre- and post-intervention data among subjects, resulting in a smaller subject group for outcomes analysis post-angioplasty. There was no control group to determine whether stent angioplasty may slow progression of FALD markers compared to patients with untreated conduit obstruction. A significant portion of the cohort had markers of significant ventricular dysfunction, low cardiac output, AVVR, and high Fontan pressures. This study utilized the MELD-XI and liver stiffness measured via ultrasound elastography as the primary surrogates for liver health, though non-invasive markers do vary center-to-center without an accepted gold standard. These markers of hepatic health are additionally influenced by the aforementioned cardiac hemodynamics. Other modalities such as magnetic resonance elastography may prove to be better at assessing FALD.

## Supplementary Information

Below is the link to the electronic supplementary material.

Supplementary file1 (DOCX 14 KB)
