# Supplementary material for: “Fontan Conduit Stent-Angioplasty and Progression of Fontan-Associated Liver Disease”
Source: Pediatr Cardiol. 2024 Mar 1;46(2):372–8. doi: 10.1007/s00246-024-03426-8 (PMC11787146; doi:10.1007/s00246-024-03426-8)
Supplement: Supplementary file 1 — Supplementary file1 (DOCX 14 KB) [file 246_2024_3426_MOESM1_ESM.docx]

***Supplemental Appendix 1***

| Palmaz 5010 stent on a 24mm x 5.5 cm BIB balloon |
| --- |
| IVC total occlusion; two Palmaz 5010 stents in addition to open cell Max LD 36mm stent placed in the IVC. 8 x 57mm Express LD iliac stent and another 8 x 37 mm Express LD iliac stent in the R common iliac, 10 x 37 mm Express LD iliac and two 9 x 25 mm express LD iliac stent in the L common and external iliac. |
| Palmaz XL biliary stent P5010, 24x5 cover mounted stent x3 |
| Fontan baffle total occlusion; 20 mm x 4 cm CP Gore-Tex covered stent 8 Zig stent with Palmaz 5010 stent on 22mm x 5.5cm BI with 24mm x 4.5cm CP Gore-Tex covered 8 Zig stent within the Palmaz stent |
| Braun Nudel 18mm x 4cm CMCPS x2 with P5010 on 18mm x 4cm BIB) |
| EV3 Max LD 35mm stent |
| 10 Zig 5 cm CP Gore-Tex covered stent |
| Cordis P4010 on 18mm x 4 cm BIB |
| EV3 Max LD 36mm stent on 20 mm x4 cm Atlas balloon |
| 8 zig CP covered stent on 25 mm BIB |
| P5010 Cordis stent on 20 mm x5.5 cm BIB |
| Palmaz 4010 stent on 18 mm x4 cm BIB |
| 8 Zig 4.5 cm CP Gore-Tex covered stent on 20 mm x5.5 cm BIB |
| NuDel 3.9 cm covered stent on 22 mm BIB |
| 14 Fr NuDel B Braun20 x 3.9 cm covered stent on 20mm balloon in balloon. |
| P4010 x2 with 20 mm x 4 cm BIB |
| 22mm x 4 cm Braun Nudel |
| NuDEL 24mm x 4.5 c, Gore-Tex CP covered stent |
| 36 mm length EV3 Max LD open cell stent |
| 8 Zig 22 mm x 3.9 cm CP pre-mounted Gore-Tex covered stent on 22mm BIB; post dilated with a 22 Atlas balloon |
| Palmaz 5010 stent on 20 x 5.5 BIB |
| BS 18mmx4cm wallstent, Cordis Palmaz 3110 stent on a 20 mm BIB |
| Braun 22mmx4cm NuDel covered stent post dilated with a 22 mm Atlas balloon |
| Braun 18mmx3.9cm NuDel covered stent x2 |
| Numed 18mmx6cm covered stent; post dilated with 20 then 22 Atlas balloons |
| NuDEL 24mmx4.5cm covered stent for Fontan then 7 mm LPA P4010 biliary stent on 18 mm balloon |
| 22mm x 4 cm Palmaz P4010 biliary stent; post dilated with 22 Atlas balloon |
| NuDel Gore-tex covered stent x2 |
| 22m x 5 cm BIB Cordis P5010 stent |
| 20mm x 5.5cm BIB Cordis P5010XL stent |
| 10 Zig 28 mm x 5 cm covered stents on 28 mm BIB |
| BBraun 26 mm x 4 cm BIB w Cordis P4010 stent; post dilated with 22mm Atlas & 25 mm Z-Med balloons |
| Nudel 8 Zig 3.9 cm CP covered stent x2; stent balloon angioplasty |
